# Factors influencing medical students’ knowledge and attitudes toward climate change: A cross-sectional study

**DOI:** 10.1371/journal.pone.0330875

**Published:** 2025-10-10

**Authors:** Leen Oyoun Alsoud, Eman Alefishat, Cynthia Al Hageh, Saif M I Alkhaaldi, Ethan Jin, Maha Al Fahim, Rasha Buhumaid, Dima Abdelmannan, Halah Ibrahim

**Affiliations:** 1 Department of Medical Sciences, Khalifa University College of Medicine and Health Sciences, Abu Dhabi, United Arab Emirates; 2 Department of Biopharmaceutics and Clinical Pharmacy, School of Pharmacy, The University of Jordan, Amman, Jordan; 3 Center for Biotechnology, Khalifa University of Science and Technology, Abu Dhabi, United Arab Emirates; 4 Department of Public Health, Khalifa University College of Medicine and Health Sciences, Abu Dhabi, United Arab Emirates; 5 Boston University, Massachusetts, United States of America; 6 Department of Medical Affairs, Sheikh Khalifa Medical City, Abu Dhabi, United Arab Emirates; 7 Department of Emergency Medicine, College of Medicine, Mohammed Bin Rashed University of Medicine and Health Sciences, Dubai Health, United Arab Emirates; 8 Department of Endocrinology, College of Medicine, Mohammed Bin Rashed University of Medicine and Health Sciences, Dubai Health, United Arab Emirates; RAK Medical and Health Sciences University, UNITED ARAB EMIRATES

## Abstract

**Introduction:**

Climate change is one of the biggest environmental challenges of the 21^st^ century. Physicians are at the forefront of recognizing, preventing, and treating climate-induced health issues. This study aims to assess attitudes, education, and knowledge of recent medical graduates regarding climate change and its health impacts, and to identify factors influencing these domains.

**Materials & methods:**

A cross-sectional web-based survey of recently graduated medical students was conducted at two large academic medical centers in the United Arab Emirates (UAE). Mean composite Likert scales were calculated. Linear regression models were utilized to study predictors of knowledge and attitude.

**Results:**

Of 458 applicants to residency programs, 311 completed the survey (67.9% response rate). Most participants were female (n=206, 66.2%), aged 25 to 30 years (n=183, 58.8%), and attended medical schools in the Middle East and North Africa (MENA) region (n=209, 67.2%). The median knowledge score was 9 out of 14 (64.3%), with an IQR of 7 to 10. The mean attitude score was 50 out of 70 (71.4%), with an IQR of 44 to 54. These results suggest moderate levels of knowledge and generally positive attitudes. Most respondents (n=197, 63.3%) did not receive climate change education in medical school. Students who completed their education in the MENA region were the least likely to have received climate change education (16.75% vs. 46.94%; *p* <.001). Survey respondents who received education demonstrated significantly improved knowledge (β=1.23, *p* <.001). Having a higher knowledge composite score was positively associated with a higher composite attitude score (β=.71, *p*=.002).

**Conclusion:**

Effects of climate change are particularly pronounced in the MENA region due to heat extremes, water scarcity, and air pollution. Recent medical graduates applying to residency programs in the UAE have had limited education in climate change. Medical schools around the world should prepare students to address the escalating health risks of climate change. This will require investing in faculty development, supporting student-led advocacy, adopting curriculum mapping tools, and most importantly, integrating clinical experience, such as project-based learning, simulations, and participatory action.

## Introduction

Climate change is one of the biggest environmental challenges of the 21st century [1]. Over 3.6 billion people are estimated to live in areas vulnerable to climate change, with 23% of global deaths attributable to environmental factors. Projections suggest that by 2030, there will be 250,000 climate change-related deaths annually [1]. Climate change can directly affect mental and physical health through severe weather events (respiratory and cardiovascular diseases, mental health disorders) and indirectly through the effects of environmental changes (vector-borne illnesses, food insecurity) [2–4]. Vulnerable populations, including young children, older adults, and low-income communities, disproportionately bear the health burdens of climate-related events [4].

Physicians are at the forefront of recognizing, preventing, and treating climate-induced health issues and serve as role models in supporting sustainable practices [5,6]. Studies show that healthcare professionals’ knowledge and attitudes toward climate change directly reflect the care they provide to their patients [7]. Although physicians should understand how environmental changes can affect human health and be comfortable counseling patients to mitigate the potential harm caused by climate change [7,8], several studies have documented a lack of knowledge and confidence in dealing with the health impacts of extreme weather events by medical students and residents [9–11]. Medical students believe neither they nor the healthcare sector are adequately prepared to address climate change [10]. Additionally, studies report that health professionals do not feel a sense of obligation to participate in climate change mitigation [12], as they perceive such efforts compromise their ability to care for their patients [13].

Worldwide, most medical schools have not formally included climate change in their curricula, relying instead on initiatives led by individual student groups or faculty members [14]. A 2020 survey demonstrated that only 15% of medical schools worldwide teach climate change [15]. A recent review of global climate change curricula found only 2 studies that described experiential learning during clinical rotations [16]. Accordingly, in a study of 600 medical students across 12 hospitals in the United States, only 6.3% felt very prepared to discuss how climate change can affect health with a patient [17]. Curricular integration is even less common in medical schools in the Global South, despite the increasing frequency and severity of climate-driven extreme weather events in these regions over the past decade [8].

The effects of climate change are particularly pronounced in the arid and semi-arid regions of the Middle East and Northern Africa (MENA), where heat extremes, water scarcity, and air pollution pose major health risks to the population [18]. Studies show an increased mortality risk in the Middle East due to circulatory events associated with increased average daily temperatures [19]. It is currently estimated that two of every three deaths from heat in the Middle East are caused by human made global warming [20]. It is projected that by the end of the century, cities around the Arabian Gulf, including Abu Dhabi and Dubai in the United Arab Emirates (UAE), could experience periods of intense heat that may make them unsuitable for human life [21]. Moreover, the MENA is among the regions with the lowest water availability in the world [18]. The water scarcity and high temperatures contribute to dust events, which increase risks of hospitalization and mortality due to respiratory and cardiovascular diseases [18]. However, there is limited published data on the attitudes, knowledge, or education of internationally educated physicians who are preparing to work in these regions. Identifying gaps in knowledge and skills can help educators and policymakers develop and implement effective educational interventions tailored to student needs [22]. To address this gap, we conducted a survey of recently graduated medical students who received education from all over the globe and were seeking residency training in the UAE. The objectives of this study were to (1) assess the knowledge, attitudes, and prior education of medical graduates regarding climate change and its health impacts, and (2) identify factors associated with higher knowledge and more positive attitudes toward climate-related health issues. We hypothesized that prior exposure to climate change education would be associated with greater knowledge and more favorable attitudes.

## Methods

A cross-sectional survey study was conducted at two large academic medical centers in the UAE. The Checklist for Reporting Results of Internet E-Surveys (CHERRIES) was used to guide our reporting [23].

### Survey development

The questionnaire was designed to assess climate change-related education in medical school curricula, participants’ knowledge of the effects of climate change on health, and their attitudes toward climate change. The survey questions were developed by a panel of medical educators and researchers after a review of the literature on climate change in medical education (S2 File). It was reviewed for content and accuracy by a public health specialist with expertise in climate change. The survey was then piloted on 15 medical students for length and clarity, with minor modifications based on their feedback. These responses were not included in the data analysis. Internal consistency of knowledge and attitude of climate change were evaluated by computing Cronbach’s alpha. Cronbach’s alpha was 0.9 for attitude and 0.6 for knowledge. The Formsite (Vroman Systems, Inc.) survey tool was used to create the survey. All questions and responses were written in English, the primary language of medical education and healthcare delivery in the UAE. Each survey question allowed only one response, which could be changed until the survey was completed and submitted. A response to each question was needed to complete the survey.

The final version of the survey (S3 File) consisted of four sections (demographics, education, knowledge, and attitude). After general demographic questions, participants were asked about formal education on climate change-related topics in medical school, with 7 questions on topics covered and 7 questions about teaching formats used. The following section included 14 multiple-choice knowledge questions on the effects of climate change on health. A cumulative score was computed by summing each participant’s responses, with a score of 1 assigned for correct answers and 0 for incorrect answers. The maximum possible cumulative score was 14, with higher scores indicating more climate change-related knowledge. The final section consisted of 14 items measuring personal attitudes to climate change. These items used a 5-point Likert-like scale ranging from strongly disagree (1) to strongly agree (5). The maximum possible score was 70, with higher scores correlating with more positive attitudes towards climate change education and sustainability practices.

### Data collection

A total population sampling approach was used, in which all applicants to residency training programs at two academic medical centers in the UAE for the 2024–2025 academic year were invited to participate. The sample size was not determined a priori through formal power calculations as the study aimed to include the entire population of eligible applicants to obtain a comprehensive assessment of their knowledge, attitudes, and education related to climate change and health. The UAE was selected as the study site because it is vulnerable to the health impacts of climate change, including extreme heat, air pollution, and water scarcity [24]. The country’s structured, competency-based medical education system attracts graduates from across the MENA region and internationally [25], making it a strategic location to assess climate change-related knowledge and attitudes among future physicians. We focused on recently graduated medical students to capture perspectives at a pivotal stage in their professional development- when they are transitioning into clinical roles and positioned to influence healthcare practice and policy. The two academic medical centers included in this study are the largest training institutions in the UAE with a large, diverse applicant pool, allowing for insights that may be transferable to other regional health systems facing similar environmental and educational challenges.

After approval by each institution’s research ethics committee, email addresses of the applicants were obtained from institution education departments. Administrators who were not involved in residency recruitment or training sent each applicant an e-mail invitation and an individual link to an online survey. The email described the purpose of the study and explained that it was anonymous and confidential. Participation was voluntary, and no incentives were offered. No IP addresses were collected for this study. The first page of the questionnaire included a consent statement. Participants who provided the written consent were able to proceed to the survey questions. Data were collected between May 6 and October 21, 2024.

### Data analysis

Data were analyzed using R (version 4.2.3). Descriptive statistics were used to measure the frequency of the variable tabulation. Linear regression models were used to measure the predictors for knowledge and attitude. Chi-squared was used to determine the associations between formal education on climate change and demographics. The assumptions of the test were evaluated. All variables included in the chi-squared tests were categorical, and expected cell counts were examined to ensure that no more than 20% of cells had expected counts less than 5. When this assumption was violated, Fisher’s exact test was used instead. For continuous variables, normality was assessed using the Shapiro-Wilk test. Normally distributed data were reported as mean ± standard deviation, while non-normally distributed data were reported as median with interquartile range (IQR). A *p*-value of <.05 was considered statistically significant. The datasets can be found in S3 File.

### Ethical approval

All methods were performed in accordance with all relevant guidelines and regulations of the Declaration of Helsinki. The study was approved by the Sheikh Khalifa Medical City Research Ethics Committee (RS-854) and the Mohammed Bin Rashid University Institutional Review Board MBRU-IRB (2024−579).

## Results

Of 458 applicants to the residency programs, 311 completed the survey (67.9% response rate). Participant demographics are presented in Table 1. Most survey respondents were female (n = 206, 66.24%), aged 25–30 years (n = 183, 58.84%), and attended medical school in the MENA region (n = 209, 67.20%). Approximately half of the respondents were applying to residency in a medical specialty (n = 172, 55.31%), and 23.8% (n = 74) were applying to surgery or a surgical specialty.

**Table 1:** Demographics of survey respondents (N = 311).

Characteristic	Participants, n (%N)
**Gender**	
Female	206 (66.24)
Male	105 (33.76)
**Age (years)**	
Under 25	114 (36.66)
25–30	183 (58.84)
31–35	11 (3.54)
Over 35	3 (0.96)
**Geographic region of medical school**	
Middle East/ North Africa	209 (67.2)
Asia	49 (15.76)
Europe	32 (10.29)
Africa	21 (6.75)
**Residency choice**	
Medical^a^	172 (55.31)
General Surgery or surgical subspecialty	74 (23.79)
Emergency medicine	30 (9.65)
Other^a^	35 (11.25)
**Received climate change education in medical school**	
Yes	78 (25.08)
No	197 (63.34)
I do not recall	36 (11.58)

^a^Medical specialties include internal medicine, family medicine, dermatology, neurology, pediatrics, and psychiatry.

^b^Other specialties include anesthesiology, radiology, and undecided.

### Climate change education received in medical school

Most respondents did not receive climate change education in their medical school curriculum (n = 197, 63.34%). Of the 78 students who did receive education, lectures were the most common teaching format (n = 70, 89.74%), followed by small group discussions (n = 44, 56.41%). Computer-based learning (n = 27, 34.62%), clerkship or clinical rotations (n = 23, 29.49%), and simulation or standardized/professional patients (n = 17, 21.79%) were rarely employed to teach climate change-related topics (Table 2). The impact of climate change on public health was the most frequently taught topic (n = 73, 93.59%), while only about half of the participants reported learning about policy implications and advocacy related to climate change and health (n = 42, 53.85%) or strategies to prepare the healthcare sector to deal with the impacts of climate change on health (n = 44; 56.41%) (Table 2).

**Table 2:** Climate change topics taught in medical school and teaching formats (N = 78).

Topic	n (%N)
Impact of climate change on public health	73 (93.59)
Basic principles of climate change	65 (83.33)
Environmental health and climate change	63 (80.77)
Effects of climate change on infectious diseases	62 (79.49)
Effects of climate change on chronic diseases	51 (65.38)
Strategies to prepare the healthcare sector to deal with the impacts of climate change on health	44 (56.41)
Policy implications and advocacy related to climate change and health	42 (53.85)
**Teaching format**	
Formal lectures	70 (89.74)
Small group discussions	44 (56.41)
Case based learning	42 (53.85)
Problem based learning	38 (48.72)
Computer based learning	27 (34.62)
Clerkships or clinical rotations	23 (29.49)
Simulation or Standardized Professional patients	17 (21.79)

### Knowledge and attitude scores

**Fig 1:**
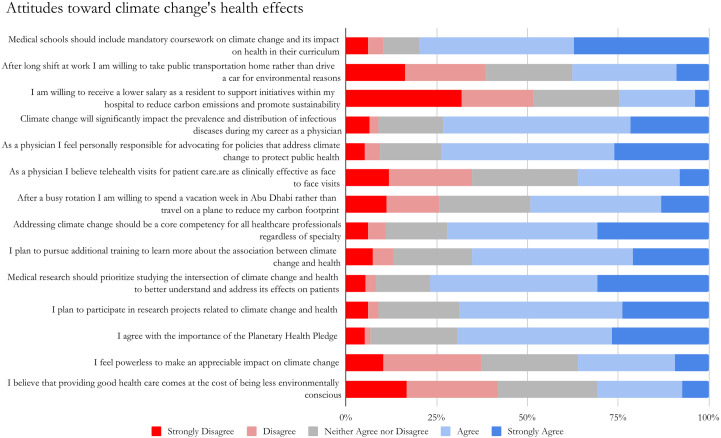
Attitude toward climate change (N = 311).

The median composite scale assessing the respondent’s knowledge was 9 out of 14 (64.3%), with an IQR of 7–10, suggesting moderate levels of knowledge. Only 77 students (24.76%) knew what their university or hospital was doing to address its carbon footprint or were familiar with the Planetary Health Pledge (n = 88, 28.30%). The mean attitude score was 50 out of 70 (71.4%), with an IQR of 44–54, suggesting generally positive attitudes. Most students agreed or strongly agreed that medical schools should include mandatory coursework on climate change and its impact on health in their curriculum (n = 248, 79.74%), felt responsible for advocating for policies that address climate change to protect public health (n = 229, 73.63%), and believed that addressing climate change should be a core competency for all healthcare professionals, regardless of specialty (n = 224; 72.03%). Respondents were also optimistic about pursuing additional training to learn more about the association between climate change and health and participating in research projects related to climate change and health (65.27% and 68.81%, respectively). Most respondents denied willingness to personally engage in behaviors to combat climate change; only 24.76% (n = 77) were willing to receive a lower salary as residents to support sustainability initiatives within their hospitals (Fig 1). Respondents who graduated from medical schools in Europe had significantly higher attitude scores than those from the MENA region (β = 4.28, *p* = .02). Also, those who chose surgery/surgical specialty as a residency specialty had significantly higher attitude composite scores than medical specialties (β = 3.05, *p* = .02) (S1 Table).

### Factors associated with knowledge and attitudes toward climate-related health issues

Chi-squared analysis was done to study the association between education level, age, gender, region of study, and residency choice (Table 3). Receiving climate change-related education was shown to be significantly associated with the study region, with medical students from Asia reporting the highest rates of receiving formal education on climate change (46.94%) while the MENA region students reported the lowest (16.75%) (*p* < .001).

**Table 3:** Chi squared analysis of receiving formal climate change education by gender, age, region, and specialty (N = 311).

Received formal climate change education	Yes, n (%N)	No, n (%N)	Do not recall, n (%N)	P-Value
**Gender**				0.06
Female	55 (26.7)	122 (59.22)	29 (14.08)	
Male	23 (21.9)	75 (71.43)	7 (6.67)	
**Age**				0.79
Under 25	29 (25.44)	71 (62.28)	14 (12.28)	
25–30	44 (24.04)	118 (64.48)	21 (11.48)	
31–35	3 (27.27)	7 (63.64)	1 (9.09)	
Over 35	2 (66.67)	1 (33.33)	0 (0)	
**Region**				<0.001
Middle East/ North Africa region	35 (16.75)	146 (69.86)	28 (13.4)	
Asia	23 (46.94)	25 (51.02)	1 (2.04)	
Europe	12 (37.5)	16 (50)	4 (12.5)	
Africa	8 (38.1)	10 (47.62)	3 (14.29)	
**Specialty**				0.68
Medical	45 (26.16)	106 (61.63)	21 (12.21)	
General surgery or surgical subspecialty	21 (28.38)	47 (63.51)	6 (8.11)	
Emergency medicine	4 (13.33)	21 (70)	5 (16.67)	
Other	8 (22.86)	23 (65.71)	4 (11.43)	

Linear regression analyzing the knowledge score predictors revealed that receiving formal education on climate change was significantly associated with a higher knowledge composite score (β = 1.23, *p* < .001) (S2 Table). Having a higher knowledge composite score was positively associated with a higher composite attitude score (β = .71, *p* = .002), suggesting that increased knowledge may contribute to shaping more positive attitudes toward the subject (S1 Table).

## Discussion

In this study of the experiences and perceptions of medical school graduates from across the globe seeking residency training in the UAE, students reported limited climate change education in medical school and minimal clinical experience managing climate change-related health conditions. Our findings are consistent with studies showing that climate change education is often absent or insufficient in medical curricula worldwide [14–16]. For example, a review of medical school curricula in the United Kingdom found wide disparities in environmental health education, ranging from 35 minutes to 9 hours of coverage [26]. Similarly, in a survey of 600 US medical students, only 13% felt that their schools provided sufficient education on climate change [17].

Survey respondents in our study also reported that traditional lectures were the primary mode of education, with minimal opportunities for experiential learning. As a result, most were entering residency without adequate preparation to care for patients affected by climate-related health conditions. This finding aligns with global studies. In one study, for instance, only 6.3% of surveyed students felt “very prepared” to discuss the impact of climate change on health [17].

A notable finding is that students from medical schools in the MENA region reported the lowest rates of formal education on climate change. These gaps in knowledge and clinical preparedness are particularly concerning given the region’s growing health challenges due to environmental change. While the MENA has adopted a National Adaptation Plan for Health and a Vulnerability and Adaptation assessment [27], and recently hosted the UN Climate Change Conference (COP 28) [28], our findings indicate that medical education has lagged behind in addressing climate-related health issues. Several factors may contribute to this gap. First, in an already crowded curriculum, emerging topics like climate change must compete with longstanding biomedical content. Additionally, a lack of faculty expertise can serve as a major barrier; many educators may not be adequately trained to deliver up-to-date, evidence-based climate health education. This challenge is further compounded by a broader lack of awareness and understanding of environmental issues across the region [29]. Moreover, medical content in the MENA region often places a stronger emphasis on traditional, discipline-based teaching, with limited interdisciplinary or public health integration [30]. The absence of national accreditation requirements that mandate climate health education may also contribute to its low prioritization [8].

One promising model for integrating climate and health education is to map relevant content onto existing curricular competencies and milestones. This can be accomplished through seminars, case-based learning, research opportunities, extracurricular activities, and clinical clerkships [7]. A recent review found that climate change-related competencies align well with established domains such as systems thinking, resource stewardship, and social justice [31]. In the US, climate and health education has evolved from isolated sessions to more coordinated, curriculum-wide approaches [32], offering useful models for embedding content into existing public health, epidemiology, or community medicine courses. This integration can help students understand the broader environmental context of diseases and health outcomes.

Future curricula should go beyond theoretical knowledge and incorporate experiential approaches that actively engage students. Clinical rotations and internships are essential for building practical skills and deepening understanding [33]. Faculty development is also crucial to support this integration. Enabling student-led advocacy has shown promise in international contexts and may offer a feasible entry point for institutions in the region.

Our study showed that although survey participants received limited formal education, they still demonstrated good knowledge regarding climate change and its health impacts. The literature on physician knowledge of climate change remains inconclusive. While some studies have identified a lack of information among medical students [34], others report that students, physicians, and clinical leaders have a good general understanding of the health implications of climate change [22,35–37]. In a survey of 837 health professions students in Jordan, nearly 90% reported the internet and social media as their primary sources of information on climate change [38]. This suggests that medical students may be independently seeking information outside formal curricula, relying on online and informal sources to fill gaps in their education- raising concerns about the consistency and credibility of these sources.

Our survey also found a positive correlation between receiving climate change education and knowledge scores. Higher knowledge was also positively correlated with more favorable attitudes toward climate change. Similarly, Abousoliman et al. found that knowledge positively correlated with attitudes toward climate change [39]. These findings suggest that curricular interventions can enhance physician attitudes toward climate change. Health professionals should be educated on balancing optimal individual care with public health promotion and sustainable practices [5,40].

Encouragingly, most survey respondents agreed that climate change should be a core competency for all health professionals and expressed a personal responsibility to advocate for policies that address climate change. This is consistent with findings from Turkey, where medical students supported integrating climate change education through both theoretical and experiential learning [41]. However, despite these positive attitudes, many respondents in our study expressed a reluctance to model sustainable practices. Fewer than half were willing to accept a lower salary as residents to support climate initiatives or take public transportation instead of driving. This hesitancy raises important questions about how medical student perceive professional responsibility and personal agency. It remains unclear whether they do not view physicians as role models for sustainability or whether they feel individual actions would have limited impact. Targeted education and training are needed to empower future physicians to take on leadership roles in promoting sustainability.

Our findings have important curricular and policy implications. The majority of physicians did not feel prepared to address the health impacts of climate change or take action. Effective education should begin early in medical training and incorporate experiential learning through project-based work, simulations, and participatory action research [42]. The current lack of a comprehensive educational framework should be addressed [7,9]. Further, collaboration between educational institutions and environmental organizations can foster new initiatives. Studies have shown that adequately training healthcare professionals enhances their role in climate advocacy [43,44] and contributes to reducing institutional, local, and national carbon emissions [17]. Medical students can also benefit from interdisciplinary approaches that bring together environmental scientists and public health experts to offer diverse perspectives.

Given the limited published research on environmental health education in the MENA region, particularly from the student perspective, our study adds to the growing international literature on climate change education. However, several limitations must be noted. Although respondents represented multiple medical schools across several countries, the sample was limited to a small number of students from two institutions, limiting generalizability. Also, only recent medical school graduates were surveyed. Future research should include a broader range of participants, including current students, residents, and faculty. Finally, the cross-sectional design reveals associations but cannot establish causality or capture changes in attitudes over time.

## Conclusions

Our study highlights the limited climate change education among newly graduated medical students applying for residency training in the UAE, reflecting global trends in medical curricula. Although students recognized climate change as an important public health issue, most reported inadequate formal education and minimal clinical exposure. Medical schools around the world must prepare students to address the growing health risks posed by climate change. This will require investments in faculty development, support for student-led advocacy, curricular mapping, and most importantly, the integration of clinical experiences, such as project-based learning, simulations, and participatory action. Interdisciplinary collaboration among medical schools, public health institutions, and environmental organizations is essential to equip future physicians with the knowledge and skills to manage climate-related health challenges and advocate for sustainable practices.

## Supporting information

S1 File Demographics of survey developers. (DOCX)

S2 File The final version of the survey. (DOCX)

S3 File The datasets. (PDF)

S1 Table Predictors of attitude score. (DOCX)

S2 Table Predictors of knowledge. (DOCX)
