# Supplementary material for: Factors influencing medical students’ knowledge and attitudes toward climate change: A cross-sectional study
Source: PLoS One. 2025 Oct 10;20(10):e0330875. doi: 10.1371/journal.pone.0330875 (PMC12513612; doi:10.1371/journal.pone.0330875)
Supplement: S1 File — (DOCX) [file pone.0330875.s001.docx]

**S1 Appendix**

**Demographics of survey developers**

|  | Role | Years of experience in role |
| --- | --- | --- |
| 1 | Clinician educator | 28 |
| 2 | Medical educator | 16 |
| 3 | Clinician educator | 10 |
| 4 | Clinician educator | 8 |
| 5 | Researcher | 7 |
| 6 | Researcher | 3 |
| 7 | Medical student | 2 |
