# Supplementary material for: Factors influencing medical students’ knowledge and attitudes toward climate change: A cross-sectional study
Source: PLoS One. 2025 Oct 10;20(10):e0330875. doi: 10.1371/journal.pone.0330875 (PMC12513612; doi:10.1371/journal.pone.0330875)
Supplement: S2 File — (DOCX) [file pone.0330875.s002.docx]

**CLIMATE CHANGE SURVEY**

We are a team of medical educators conducting a study on medical trainee knowledge and training in climate change. The purpose of this study is to understand the awareness and preparedness of the next generation of healthcare professionals regarding climate change and its effects on health. Your responses will help us to create an educational program for medical trainees. All data will be analyzed in aggregate. You or your institution cannot be identified in any way. Your participation is voluntary. You will not receive any compensation for participating. The survey will take approximately 5 minutes to complete. Thank you in advance for your participation.

- I agree to participate in the research study.

**SECTION A DEMOGRAPHICS**

What is your gender?

1. Male
2. Female

What is your age?

1. Under 25
2. 25-30
3. 31-35
4. Over 35

In what region did you attend medical school?

1. Middle East/ North Africa region
2. Africa
3. Asia
4. Australia/New Zealand
5. Europe
6. North America
7. South America

What specialty are you pursuing in your residency (first choice)?

1. Dermatology
2. Emergency medicine
3. Family medicine
4. General Surgery or surgical subspecialty
5. Internal medicine or medical subspecialty
6. Neurology
7. Obstetrics/Gynecology
8. Pediatrics
9. Psychiatry
10. Radiology
11. Other

**SECTION B EDUCATION**

During medical school, did you receive any formal teaching on climate change and its health effects?

1. Yes
2. No
3. I do not recall

If yes, which of the following topics were covered?

Basic principles of climate change

1. Yes
2. No
3. I do not recall

Impact of climate change on public health

1. Yes
2. No
3. I do not recall

Effects of climate change on infectious diseases

1. Yes
2. No
3. I do not recall

Effects of climate change on chronic diseases

1. Yes
2. No
3. I do not recall

Environmental health and climate change

1. Yes
2. No
3. I do not recall

Strategies to prepare the healthcare sector to deal with the impacts of climate change on health

1. Yes
2. No
3. I do not recall

Policy implications and advocacy related to climate change and health

1. Yes
2. No
3. I do not recall

Were the following teaching formats used for climate change education?

Formal lectures

1. Yes

2. No

3. I do not recall

Case-based learning

1. Yes

2. No

3. I do not recall

Small group discussions

1. Yes

2. No

3. I do not recall

Problem-based learning

1. Yes

2. No

3. I do not recall

Computer-based learning

1. Yes

2. No

3. I do not recall

Clerkships or clinical rotations

1. Yes

2. No

3. I do not recall

Simulation or Standardized/Professional patients

1. Yes

2. No

3. I do not recall

**SECTION C AWARENESS**

I am knowledgeable about how to address climate change with my patients

1. Yes
2. No
3. Not sure

I am aware of what my university or hospital is doing to address its carbon footprint

1. Yes
2. No
3. Not sure

I am aware of the Planetary Health Pledge

1. Yes
2. No
3. N. ot sure

Medication-prescribing practices have the potential to substantially reduce waste and the associated carbon footprint

1. Yes
2. No
3. Not sure

**Answer:** 1) Yes

**SECTION D KNOWLEDGE**

The health sector’s contribution to global emissions is

1. 4-5%
2. 10-20%
3. 25-30%
4. 50%

**Answer:** 1) 4-5%

Which of the following actions can individuals take to mitigate climate change and its health impacts?

1. Consuming more red meat
2. Driving alone in a gas-guzzling car
3. Using energy-efficient appliances
4. Increasing air travel for leisure purposes

**Answer:** 3) Using energy-efficient appliances

How does climate change contribute to the spread of infectious diseases?

1. Reducing habitat loss for disease vectors
2. Increasing the availability of clean water sources
3. Promoting the survival and spread of disease-carrying insects
4. Decreasing the temperature suitable for bacterial growth

**Answer:** 3) Promoting the survival and spread of disease-carrying insects

Which of the following are associated with greater heat-related morbidity and mortality?

1. Older age
2. Diabetes mellitus
3. Pregnancy
4. All of the above

**Answer:** 4) All of the above

Exposure to air pollution can lead to cardiovascular problems:

1. Only after long-term exposure
2. After either long- or short-term exposure
3. Only in people at risk
4. Only in individuals with Type 2 Diabetes Mellitus

**Answer:** 2) After either long- or short-term exposure

Which of the following statements about heart failure and heat exposure is true?

1. Heat exposure does not affect heart failure symptoms
2. Medications to treat heart failure can increase the risk of harm from heat exposure
3. Patients with heart failure should exercise in the heat
4. Diuretics may decrease the risk of harm during heat exposure

**Answer:** 2) Medications to treat heart failure increase the risk of harm from heat exposure

Which population group is most vulnerable to the health impacts of climate change?

1. Young adults between ages 20 and 25
2. Older adults above age 65
3. Athletes
4. Vegetarians

**Answer:** 2) Older adults

Which of the following is a potential consequence of climate change on mental health?

1. Increased rates of depression
2. Reduced stigma surrounding mental illness
3. Enhanced social support networks
4. Improved coping mechanisms

**Answer:** 1) Increased rates of depression

Survivors of hurricanes, wildfires or other weather-related disasters have an increased risk of which of the following?

1. Respiratory infections
2. Post-traumatic stress disorder (PTSD)
3. Myocardial infarctions
4. All of the above

**Answer:** 4) All of the above

Which of the following is an example of a climate change adaptation strategy relevant to healthcare?

1. Designing hospitals to increase energy and water consumption
2. Ignoring the potential health impacts of climate change
3. Developing emergency preparedness plans for extreme weather events
4. Relying solely on fossil fuels for energy needs

**Answer:** 3) Developing emergency preparedness plans for extreme weather events

**SECTION E ATTITUDES**

Please use the following scale to indicate your level of agreement or disagreement with each statement. (1=strongly disagree; 2=disagree; 3=neither agree nor disagree; 4=agree; 5=strongly agree)

1. Medical schools should include mandatory coursework on climate change and its impact on health in their curriculum.
2. After a long shift at work, I am willing to take public transportation home rather than drive a car for environmental reasons.
3. I am willing to receive a lower salary as a resident to support initiatives within my hospital to reduce carbon emissions and promote sustainability
4. Climate change will significantly impact the prevalence and distribution of infectious diseases during my career as a physician.
5. As a physician, I feel personally responsible for advocating for policies that address climate change to protect public health.
6. As a physician, I believe telehealth visits for patient care are as clinically effective as face-to-face visits
7. After a busy rotation, I am willing to spend a vacation week in Abu Dhabi rather than travel on a plane to reduce my carbon footprint
8. Addressing climate change should be a core competency for all healthcare professionals, regardless of specialty.
9. I plan to pursue additional training to learn more about the association between climate change and health.
10. Medical research should prioritize studying the intersection of climate change and health to better understand and address its effects on patients.
11. I plan to participate in research projects related to climate change and health.
12. I agree with the importance of the Planetary Health Pledge
13. I feel powerless to make an appreciable impact on climate change
14. I believe that providing good health care comes at the cost of being less environmentally conscious
