# Supplementary material for: Factors influencing medical students’ knowledge and attitudes toward climate change: A cross-sectional study
Source: PLoS One. 2025 Oct 10;20(10):e0330875. doi: 10.1371/journal.pone.0330875 (PMC12513612; doi:10.1371/journal.pone.0330875)
Supplement: S3 File — (PDF) [file pone.0330875.s003.pdf]

attitude co	education	gender	age	region	specialty	knowledge composite
54	Yes	Female	Over 35	Asia	medical	12
52	No	Female	Under 25	Europe	Emergency	10
48	No	Male	25-30	Asia	General Su	10
60	No	Male	25-30	Asia	medical	8
41	No	Female	Under 25	Middle Eas	General Su	7
58	Yes	Male	Under 25	Middle Eas	General Su	14
50	No	Male	25-30	Middle Eas	medical	9
60	Yes	Male	Under 25	Middle Eas	General Su	12
53	No	Male	25-30	Europe	medical	10
57	No	Male	25-30	Middle Eas	General Su	9
50	No	Female	25-30	Africa	medical	10
62	Yes	Male	Under 25	Middle Eas	medical	14
47	No	Female	Under 25	Middle Eas	medical	5
59	No	Female	Under 25	Middle Eas	medical	7
43	Yes	Female	25-30	Asia	medical	11
46	No	Male	25-30	Middle Eas	General Su	7
57	No	Male	25-30	Middle Eas	medical	7
41	No	Male	Under 25	Middle Eas	General Su	8
62	No	Male	25-30	Middle Eas	medical	8
58	No	Male	25-30	Asia	General Su	7
56	No	Male	25-30	Africa	Emergency	8
61	No	Male	25-30	Asia	General Su	8
55	No	Female	Under 25	Middle Eas	medical	8
63	No	Male	25-30	Africa	Emergency	9
61	No	Male	25-30	Asia	General Su	8
52	No	Female	25-30	Europe	General Su	10
59	No	Male	25-30	Middle Eas	medical	9
58	No	Male	25-30	Middle Eas	medical	11
62	No	Male	25-30	Asia	medical	8
59	No	Male	25-30	Asia	General Su	8
56	No	Female	25-30	Africa	General Su	7
35	No	Female	Under 25	Middle Eas	General Su	3
60	No	Female	Under 25	Middle Eas	General Su	11
55	No	Female	25-30	Middle Eas	General Su	7
54	Yes	Female	Under 25	Middle Eas	General Su	13
62	No	Male	25-30	Middle Eas	General Su	8
69	Yes	Female	Under 25	Middle Eas	General Su	14
63	No	Male	25-30	Middle Eas	General Su	8
61	No	Male	25-30	Asia	medical	8
59	No	Male	25-30	Asia	General Su	8
62	Yes	Female	25-30	Middle Eas	General Su	13
44	No	Female	25-30	Middle Eas	General Su	3
50	I do not rec	Female	25-30	Middle Eas	General Su	11
57	No	Female	Under 25	Middle Eas	General Su	6
52	No	Male	25-30	Africa	medical	6
41	No	Female	Under 25	Middle Eas	General Su	9
50	I do not rec	Female	Under 25	Middle Eas	General Su	8
40	No	Female	25-30	Middle Eas	General Su	9
40	No	Female	31-35	Middle Eas	medical	6

41	No	Female	25-30	Middle Eas	medical	9
42	No	Male	25-30	Middle Eas	medical	9
38	No	Female	25-30	Middle Eas	medical	10
35	No	Female	25-30	Middle Eas	medical	8
63	Yes	Female	Under 25	Europe	medical	10
48	No	Male	Under 25	Middle Eas	medical	8
48	I do not rec	Female	25-30	Middle Eas	medical	10
36	No	Female	25-30	Middle Eas	medical	10
39	No	Female	25-30	Middle Eas	medical	5
39	No	Male	Under 25	Middle Eas	Emergency	7
54	No	Female	25-30	Europe	General Su	6
55	Yes	Female	Under 25	Middle Eas	medical	8
39	I do not rec	Male	25-30	Middle Eas	medical	8
37	No	Female	25-30	Middle Eas	medical	11
53	No	Male	25-30	Middle Eas	medical	11
45	No	Female	25-30	Middle Eas	medical	8
49	No	Female	25-30	Europe	medical	10
52	No	Female	Under 25	Middle Eas	other	10
23	Yes	Male	Under 25	Asia	General Su	11
48	Yes	Male	Under 25	Asia	Emergency	11
51	I do not rec	Female	Under 25	Middle Eas	General Su	6
50	No	Female	Under 25	Europe	Emergency	13
62	Yes	Female	Under 25	Europe	medical	12
54	I do not rec	Male	Under 25	Middle Eas	medical	11
26	I do not rec	Male	25-30	Middle Eas	medical	7
49	Yes	Female	25-30	Middle Eas	medical	6
52	No	Female	25-30	Middle Eas	medical	8
24	Yes	Female	Under 25	Asia	medical	9
51	No	Female	25-30	Middle Eas	medical	10
47	No	Male	Under 25	Middle Eas	General Su	7
59	No	Female	31-35	Middle Eas	medical	11
52	Yes	Male	25-30	Africa	medical	7
50	No	Female	Under 25	Middle Eas	Emergency	6
43	No	Female	25-30	Middle Eas	medical	10
38	No	Male	Under 25	Middle Eas	General Su	9
43	I do not rec	Female	25-30	Middle Eas	medical	11
42	No	Male	25-30	Middle Eas	medical	6
22	Yes	Female	Under 25	Middle Eas	medical	12
51	No	Male	Under 25	Middle Eas	medical	12
22	Yes	Female	25-30	Middle Eas	medical	13
51	I do not rec	Female	Under 25	Europe	medical	11
39	Yes	Male	25-30	Middle Eas	medical	10
46	No	Female	25-30	Middle Eas	other	5
48	Yes	Female	31-35	Africa	medical	10
58	Yes	Female	25-30	Asia	other	9
53	Yes	Female	25-30	Asia	other	8
46	No	Female	25-30	Europe	medical	6
41	No	Male	25-30	Middle Eas	medical	13
29	No	Male	25-30	Middle Eas	medical	9
23	I do not rec	Female	25-30	Middle Eas	medical	3

52	No	Female	25-30	Middle Eas	medical	6
49	No	Female	25-30	Middle Eas	medical	11
22	Yes	Female	Under 25	Asia	medical	9
60	No	Male	25-30	Asia	medical	10
55	Yes	Female	25-30	Africa	medical	9
41	I do not rec	Male	Under 25	Middle Eas	medical	5
44	No	Male	25-30	Middle Eas	medical	5
50	No	Female	25-30	Middle Eas	other	6
52	Yes	Female	25-30	Middle Eas	medical	11
48	No	Male	25-30	Asia	medical	7
50	No	Male	25-30	Asia	General Su	7
38	Yes	Female	25-30	Middle Eas	General Su	9
45	I do not rec	Female	Under 25	Middle Eas	medical	8
52	No	Female	25-30	Africa	other	8
49	Yes	Male	25-30	Europe	other	9
43	No	Male	31-35	Middle Eas	medical	8
49	No	Male	25-30	Asia	Emergency	9
48	Yes	Male	25-30	Asia	General Su	9
52	No	Female	Under 25	Middle Eas	General Su	4
50	No	Female	25-30	Middle Eas	medical	10
55	Yes	Female	25-30	Middle Eas	medical	7
56	No	Female	25-30	Middle Eas	medical	13
48	No	Female	Under 25	Middle Eas	medical	9
40	No	Female	25-30	Middle Eas	medical	9
46	I do not rec	Female	Under 25	Middle Eas	medical	6
58	I do not rec	Female	Under 25	Middle Eas	medical	7
40	Yes	Male	31-35	Middle Eas	medical	5
63	No	Female	25-30	Asia	medical	6
54	No	Female	Under 25	Asia	medical	3
54	No	Female	25-30	Africa	medical	6
52	No	Male	Under 25	Middle Eas	other	5
45	No	Female	31-35	Asia	medical	5
22	No	Female	25-30	Middle Eas	medical	3
22	No	Male	Under 25	Middle Eas	other	3
62	No	Female	25-30	Europe	medical	7
52	Yes	Male	25-30	Middle Eas	medical	7
33	Yes	Female	25-30	Africa	other	12
62	No	Female	25-30	Middle Eas	medical	10
61	No	Female	31-35	Middle Eas	medical	10
48	Yes	Male	25-30	Europe	medical	9
47	No	Male	31-35	Middle Eas	Emergency	11
51	No	Female	25-30	Middle Eas	medical	10
57	No	Female	Under 25	Middle Eas	medical	10
42	No	Male	Under 25	Middle Eas	other	7
52	No	Female	Under 25	Middle Eas	medical	11
53	I do not rec	Female	25-30	Africa	other	8
49	I do not rec	Female	25-30	Middle Eas	other	9
22	Yes	Female	25-30	Asia	other	9
52	I do not rec	Female	25-30	Europe	medical	11
51	No	Female	25-30	Europe	medical	10

23	I do not rec	Male	25-30	Middle Eas	Emergency	3
22	No	Female	Under 25	Middle Eas	Emergency	8
50	I do not rec	Female	Under 25	Middle Eas	General Su	7
48	Yes	Female	25-30	Asia	other	5
39	Yes	Female	Under 25	Middle Eas	General Su	4
51	No	Female	25-30	Middle Eas	Emergency	10
46	No	Male	25-30	Asia	General Su	7
48	Yes	Male	25-30	Asia	medical	9
50	No	Male	Under 25	Middle Eas	General Su	8
47	No	Male	Under 25	Middle Eas	Emergency	8
57	No	Male	Under 25	Middle Eas	Emergency	9
49	I do not rec	Female	Under 25	Middle Eas	Emergency	8
53	I do not rec	Female	25-30	Middle Eas	medical	10
45	No	Male	25-30	Middle Eas	medical	10
52	I do not rec	Female	25-30	Middle Eas	General Su	4
45	Yes	Male	25-30	Asia	General Su	9
57	Yes	Female	Under 25	Middle Eas	medical	8
46	No	Female	Under 25	Middle Eas	other	11
49	No	Male	25-30	Middle Eas	General Su	7
48	No	Female	25-30	Middle Eas	medical	4
34	No	Female	Under 25	Middle Eas	medical	7
53	Yes	Female	25-30	Middle Eas	medical	14
58	No	Male	Under 25	Middle Eas	medical	7
41	No	Female	Under 25	Middle Eas	medical	11
58	No	Male	Under 25	Middle Eas	medical	9
45	No	Female	25-30	Middle Eas	medical	8
59	Yes	Female	25-30	Asia	General Su	9
49	No	Male	25-30	Middle Eas	Emergency	11
46	I do not rec	Male	25-30	Middle Eas	medical	9
50	Yes	Female	Under 25	Asia	medical	10
52	No	Male	25-30	Middle Eas	medical	10
53	Yes	Female	25-30	Europe	medical	8
41	No	Male	Under 25	Middle Eas	medical	10
52	Yes	Female	Under 25	Middle Eas	medical	8
46	I do not rec	Female	25-30	Middle Eas	medical	8
38	Yes	Female	25-30	Middle Eas	medical	6
65	Yes	Female	Under 25	Middle Eas	medical	7
59	No	Female	Under 25	Middle Eas	medical	8
49	Yes	Male	25-30	Africa	medical	9
43	No	Female	25-30	Middle Eas	Emergency	6
47	Yes	Female	Under 25	Middle Eas	medical	10
58	Yes	Female	Under 25	Middle Eas	medical	13
53	No	Female	Under 25	Middle Eas	medical	7
47	I do not rec	Female	Under 25	Middle Eas	medical	5
41	I do not rec	Female	25-30	Middle Eas	medical	5
43	I do not rec	Male	Under 25	Middle Eas	medical	4
31	No	Female	25-30	Middle Eas	other	11
45	No	Female	Under 25	Middle Eas	medical	10
50	No	Female	Under 25	Middle Eas	medical	7
52	No	Female	25-30	Middle Eas	medical	10

50	No	Female	25-30	Europe	medical	9
58	Yes	Female	25-30	Middle Eas	medical	10
44	No	Male	Under 25	Middle Eas	other	10
57	Yes	Male	25-30	Africa	other	10
52	No	Male	Under 25	Middle Eas	Emergency	8
62	No	Female	Under 25	Middle Eas	Emergency	8
40	No	Male	25-30	Africa	medical	10
55	I do not rec	Female	Under 25	Middle Eas	Emergency	9
58	No	Female	Under 25	Europe	medical	9
53	No	Female	Under 25	Middle Eas	Emergency	8
60	No	Female	25-30	Asia	other	8
64	No	Male	25-30	Asia	other	9
50	No	Male	Under 25	Middle Eas	medical	8
62	No	Female	25-30	Middle Eas	General Su	8
51	No	Female	Under 25	Middle Eas	medical	9
48	No	Female	Under 25	Middle Eas	medical	9
52	No	Male	25-30	Asia	medical	14
46	Yes	Female	25-30	Middle Eas	Emergency	8
54	Yes	Female	Under 25	Asia	medical	9
55	Yes	Male	Under 25	Middle Eas	medical	13
52	No	Female	Under 25	Middle Eas	General Su	6
49	No	Female	Under 25	Middle Eas	medical	11
54	I do not rec	Female	25-30	Europe	medical	6
52	Yes	Male	25-30	Africa	medical	10
62	Yes	Female	Under 25	Middle Eas	medical	11
42	I do not rec	Female	Under 25	Middle Eas	General Su	8
52	No	Female	25-30	Middle Eas	medical	11
49	No	Female	25-30	Middle Eas	medical	8
52	No	Female	Under 25	Europe	medical	7
59	No	Female	Under 25	Middle Eas	General Su	11
52	No	Female	Under 25	Middle Eas	General Su	11
41	No	Female	25-30	Middle Eas	General Su	4
59	No	Female	Under 25	Middle Eas	General Su	8
54	Yes	Female	Under 25	Asia	General Su	5
49	No	Male	Under 25	Middle Eas	medical	4
45	Yes	Female	25-30	Asia	medical	13
52	No	Female	Under 25	Middle Eas	other	8
25	Yes	Female	25-30	Africa	General Su	7
47	No	Male	25-30	Africa	medical	9
42	No	Female	25-30	Middle Eas	Emergency	4
52	Yes	Female	25-30	Europe	medical	9
50	I do not rec	Female	25-30	Africa	medical	10
47	Yes	Male	Under 25	Europe	medical	5
48	No	Female	25-30	Europe	medical	8
45	Yes	Female	Under 25	Europe	General Su	10
46	I do not rec	Female	25-30	Asia	medical	9
31	No	Female	25-30	Asia	medical	4
43	No	Male	25-30	Asia	medical	11
52	No	Female	25-30	Middle Eas	medical	10
60	No	Female	Under 25	Middle Eas	General Su	8

22	No	Male	Over 35	Middle Eas	medical	6
46	No	Female	Under 25	Middle Eas	other	9
40	No	Female	25-30	Middle Eas	medical	3
42	No	Female	Under 25	Middle Eas	other	2
39	No	Male	Under 25	Middle Eas	other	9
62	Yes	Female	25-30	Europe	General Su	5
45	Yes	Male	25-30	Asia	General Su	7
49	No	Male	31-35	Middle Eas	medical	7
66	Yes	Male	Under 25	Middle Eas	Emergency	10
60	Yes	Female	25-30	Europe	General Su	9
42	Yes	Female	Under 25	Middle Eas	medical	10
50	No	Female	25-30	Middle Eas	General Su	6
47	I do not rec	Female	31-35	Europe	Emergency	8
40	I do not rec	Female	25-30	Middle Eas	other	9
56	Yes	Female	25-30	Asia	General Su	13
38	No	Female	25-30	Middle Eas	medical	10
44	No	Male	Under 25	Middle Eas	medical	9
52	No	Female	25-30	Middle Eas	medical	5
44	Yes	Male	25-30	Middle Eas	Emergency	6
50	No	Female	Under 25	Middle Eas	Emergency	6
42	No	Female	Under 25	Middle Eas	medical	6
42	No	Female	25-30	Africa	medical	7
30	No	Female	25-30	Middle Eas	General Su	11
43	No	Male	25-30	Middle Eas	General Su	10
53	No	Female	Under 25	Middle Eas	other	10
47	No	Female	25-30	Middle Eas	medical	9
45	No	Male	25-30	Middle Eas	other	9
37	No	Female	25-30	Europe	medical	10
59	I do not rec	Female	25-30	Africa	other	8
51	No	Female	Under 25	Middle Eas	medical	7
48	Yes	Female	25-30	Asia	medical	7
47	No	Female	25-30	Asia	medical	6
25	No	Male	25-30	Middle Eas	Emergency	10
46	No	Female	Under 25	Middle Eas	other	7
44	No	Male	25-30	Middle Eas	General Su	7
45	No	Female	Under 25	Middle Eas	other	9
51	Yes	Female	25-30	Europe	medical	11
55	No	Female	25-30	Middle Eas	General Su	8
44	No	Female	25-30	Middle Eas	General Su	10
49	No	Female	Under 25	Middle Eas	medical	7
51	Yes	Female	25-30	Asia	medical	10
43	No	Male	25-30	Middle Eas	medical	6
52	No	Female	25-30	Middle Eas	medical	9
46	No	Female	25-30	Middle Eas	medical	6
37	Yes	Male	31-35	Middle Eas	medical	8
30	Yes	Female	25-30	Asia	medical	9
61	No	Female	25-30	Middle Eas	General Su	5
54	Yes	Female	25-30	Middle Eas	General Su	8
58	Yes	Female	25-30	Europe	General Su	9
47	No	Male	Under 25	Middle Eas	other	10

63	No	Male	25-30	Asia	General Su	12
63	No	Female	25-30	Middle Eas	medical	7
52	No	Female	25-30	Middle Eas	Emergency	11
49	No	Female	25-30	Asia	other	8
60	No	Female	25-30	Europe	General Su	12
36	No	Female	25-30	Europe	other	11
41	No	Female	Under 25	Middle Eas	General Su	9
48	I do not rec	Female	25-30	Middle Eas	Emergency	8
38	No	Male	Under 25	Middle Eas	medical	8
47	Yes	Female	Over 35	Middle Eas	other	9
60	Yes	Female	Under 25	Middle Eas	General Su	9
48	No	Female	25-30	Middle Eas	General Su	11
