# Supplementary material for: Factors influencing medical students’ knowledge and attitudes toward climate change: A cross-sectional study
Source: PLoS One. 2025 Oct 10;20(10):e0330875. doi: 10.1371/journal.pone.0330875 (PMC12513612; doi:10.1371/journal.pone.0330875)
Supplement: S1 Table — (DOCX) [file pone.0330875.s004.docx]

**S1 Table:**

| **S1 Table.** Predictors of attitude score. | | | | |
| --- | --- | --- | --- | --- |
| Characteristic | Estimate | Std. Error | t value | Pr(>\|t\|) |
| **(Intercept)** | 42.74 | 2.21 | 19.31 | 0 |
| **Education** |  |  |  |  |
| Yes | -1.61 | 1.32 | -1.22 | 0.22 |
| No | 0.00 | - | - | - |
| I do not recall | -1.98 | 1.68 | -1.18 | 0.24 |
| **Gender** |  |  |  |  |
| Female | 0.00 | - | - | - |
| Male | -0.37 | 1.14 | -0.32 | 0.75 |
| **Age** |  |  |  |  |
| Under 25 | 0.00 | - | - | - |
| 25-30 | -1.59 | 1.16 | -1.37 | 0.17 |
| 31-35 | -0.13 | 2.87 | -0.05 | 0.96 |
| Over 35 | -5.64 | 5.37 | -1.05 | 0.29 |
| **Region** |  |  |  |  |
| Middle East/ North Africa region | 0.00 | - | - | - |
| Asia | 2.31 | 1.57 | 1.47 | 0.14 |
| Europe | 4.28 | 1.78 | 2.4 | 0.02 |
| Africa | 3.64 | 2.21 | 1.65 | 0.1 |
| **Specialty** |  |  |  |  |
| Medical specialty | 0.00 | - | - | - |
| General Surgery or surgical subspecialty | 3.05 | 1.29 | 2.36 | 0.02 |
| Emergency medicine | -0.11 | 1.83 | -0.06 | 0.95 |
| Other | -1.52 | 1.72 | -0.89 | 0.38 |
| **Knowledge and familiarity score** | 0.71 | 0.23 | 3.12 | 0.002 |
