# Supplementary material for: Factors influencing medical students’ knowledge and attitudes toward climate change: A cross-sectional study
Source: PLoS One. 2025 Oct 10;20(10):e0330875. doi: 10.1371/journal.pone.0330875 (PMC12513612; doi:10.1371/journal.pone.0330875)
Supplement: S2 Table — (DOCX) [file pone.0330875.s005.docx]

S2 Table

| **S2 Table**. Predictors of knowledge and familiarity. | | | | |
| --- | --- | --- | --- | --- |
| Characteristic | Estimate | Std. Error | t value | Pr(>\|t\|) |
| **(Intercept)** | 6.06 | 0.75 | 8.09 | 0.001 |
| **Education** |  |  |  |  |
| Yes | 1.23 | 0.32 | 3.84 | <0.001 |
| No | 0.00 | - | - | - |
| I do not recall | -0.40 | 0.42 | -0.96 | 0.34 |
| **Gender** |  |  |  |  |
| Female | 0.00 | - | - | - |
| Male | 0.23 | 0.28 | 0.81 | 0.42 |
| **Age** |  |  |  |  |
| Under 25 | 0.00 | - | - | - |
| 25-30 | 0.09 | 0.29 | 0.31 | 0.75 |
| 31-35 | -0.43 | 0.73 | -0.6 | 0.55 |
| Over 35 | 0.36 | 1.34 | 0.26 | 0.79 |
| **Region** |  |  |  |  |
| Middle East/ North Africa region | 0.00 | - | - | - |
| Asia | -0.26 | 0.39 | -0.66 | 0.51 |
| Europe | 0.31 | 0.45 | 0.69 | 0.49 |
| Africa | -0.20 | 0.55 | -0.35 | 0.72 |
|  |  |  |  |  |
| **Specialty** |  |  |  |  |
| Medical | 0.00 | - | - | - |
| General surgery or surgical subspecialty | -0.42 | 0.32 | -1.3 | 0.2 |
| Emergency medicine | -0.03 | 0.46 | -0.07 | 0.94 |
| Other | -0.14 | 0.43 | -0.33 | 0.74 |
| **Attitude score** | 0.04 | 0.01 | 3.12 | 0.002 |
